# Evaluation of a Novel 3D-Printed Urinary Catheterization Simulation Model in Undergraduate Medical Teaching

**DOI:** 10.7759/cureus.8377

**Published:** 2020-05-31

**Authors:** Charlie J Gillis, Nicole Bishop, Gregory Walsh, David Harvey

**Affiliations:** 1 Urology, Dalhousie University, Halifax, CAN; 2 Medical Education and Simulation, Memorial University of Newfoundland, St. John's, CAN; 3 Memorial University of Newfoundland (MUN) Med 3d Laboratory, Memorial University of Newfoundland and Labrador, St. John's, CAN; 4 Urology, Memorial University of Newfoundland and Labrador, St. John's, CAN

**Keywords:** urinary catheter, 3d-printing, simulation, medical education

## Abstract

Introduction

Urinary catheter insertion is a mandatory procedure taught during medical school. It is imperative that learners are provided the opportunity to practice the procedure, as an improper catheterization technique can result in urethral trauma and contribute to urinary tract infections. Simulation training offers the advantage of avoiding patient harm while allowing learners to feel comfortable to learn from their mistakes, resulting in increased user confidenceand shortening the learning curve for basic procedures. 3D-printed simulation models are anatomically accurate, low-cost, reusable, and effective for teaching basic procedural skills.This study aims to assess the self-rated effectiveness of the 3D model in increasing student confidence and preparedness.

Methods

Preclerkship undergraduate medical students (n=64) participated in procedural skills training sessions using the 3D-printed model. The students were provided with didactic teaching from a urologist, a hands-on demonstration, and then allowed to practice the procedure using the 3D model. Students were subsequently asked to complete a Likert-type survey to evaluate their experience and the 3D model as an educational tool.

Results

Respondents felt that the 3D-printed model allowed for the realistic and accurate performance of a urinary catheterization procedure, allowing students to increase their confidence, competence, and knowledge of the technique. Student responses for increasing competence were rated as an average of 4.48±0.62 (where 1 is “not at all effective” and 5 is “very effective”), confidence was rated as 4.40±0.71, and preparedness was reported as 4.15±0.76. Overall, the reported value as a training tool resulted in an average score of 4.62±0.58 (where 1 is “not at all relevant” and 5 is “very relevant”).

Conclusions

Preclerkship undergraduate medical students found the 3D-printed male catheter insertion model to be a useful learning tool with accurate anatomical representations and technical qualities. The 3D-printed model can be beneficial for increasing learner confidence and preparedness when completing a catheter insertion, allowing for the opportunity to practice on a low-cost, accessible simulator.

## Introduction

Urinary catheterization is performed extensively in acute care centers in Canada for fluid status assessment, for specific genitourinary procedures, or for those patients unable to control their bladder function and void independently. Despite the prevalence of catheters, a large portion of iatrogenic urethral injury is related to their insertion by junior learners [1]. An improper insertion technique can result in bleeding, false passage, sepsis, and stricture, sequelae that have the potential for significant mortality and lifelong morbidity [1-2]. An incorrect insertion technique is found to be related to the development of catheter-associated urinary tract infections (UTIs), and the preservation of sterility during the initial insertion procedure has the potential to reduce the incidence of these infections [3-4]. These iatrogenic complications are found to be avoidable with the proper insertion technique, and catheter insertion training programs have been shown to minimize the potential for injury [5].

Given the increasing focus on optimal patient outcomes and the greater risk of injury as a procedure is being learned initially, simulation-based training is evolving to allow learners to make mistakes in a safe environment [6]. Not only does this result in improved patient safety, but simulation has been shown to shorten the learning curve to technical proficiency in a variety of procedures [7]. Simulation allows for the acquisition of basic technical skills while remaining safe, cost-efficient, and time-efficient [8]. Specific to urology, simulation is particularly effective for easily emulated endourological and laparoscopic procedures, yet it is also useful for performing and visualizing more basic genitourinary techniques like catheterization and digital rectal examination (DRE) [9-12]. Catheter simulation allows learners to familiarize themselves with the technical aspects of the procedure without the added stress of performing it on a real patient for the first time.

Simulation using 3D-printed models has the advantage of being easily produced, inexpensive, with transferrable designs, and highly anatomically correct [13-15]. Specific patient anatomy can be incorporated using contemporary imaging techniques, allowing for the appreciation of individual anatomy for surgical planning or as a patient teaching aid. Designs can be shared between sites, allowing remote teaching sites to easily produce simulation models prior to the point of care. For the acquisition of basic procedural skills, low-fidelity models have been shown to be as effective as high-fidelity models, emphasizing the advantages of rapidly produced, inexpensive 3D-printed simulation models [16].

Catheter insertion is a hands-on skill typically taught to medical students in pre-clerkship simulation training, allowing for evaluation during the clinical clerkship rotations [17]. Medical students have been shown to benefit from urinary catheterization simulation training and can achieve similar patient outcomes to those of other health care professionals [18]. Despite the capability of medical students to achieve clinical excellence in this technique and the necessity of having a basic clinical understanding of the indications and risks of catheter insertion for the patients they will be attending, many students report low levels of confidence in their ability to perform this procedure [19-20]. Medical students have been implicated in the development of catheter-associated urinary tract infections, emphasizing the need for consistent hands-on and simulation training to help promote a sterile technique and prevent these sequelae [21]. Participating in simulation training can significantly improve medical student confidence in urinary catheterization, and this training is highly rated by students as being useful in their medical education [9,20,22].

A 3D-printed urinary catheterization model has the potential to provide useful anatomical and procedural simulation training for pre-clerkship medical students before performing this technique on a patient during their clerkship years. This article describes the development of a 3D-printed simulation model and novice feedback from pre-clerkship students learning catheterization on this model.

## Materials and methods

**Figure 1:**
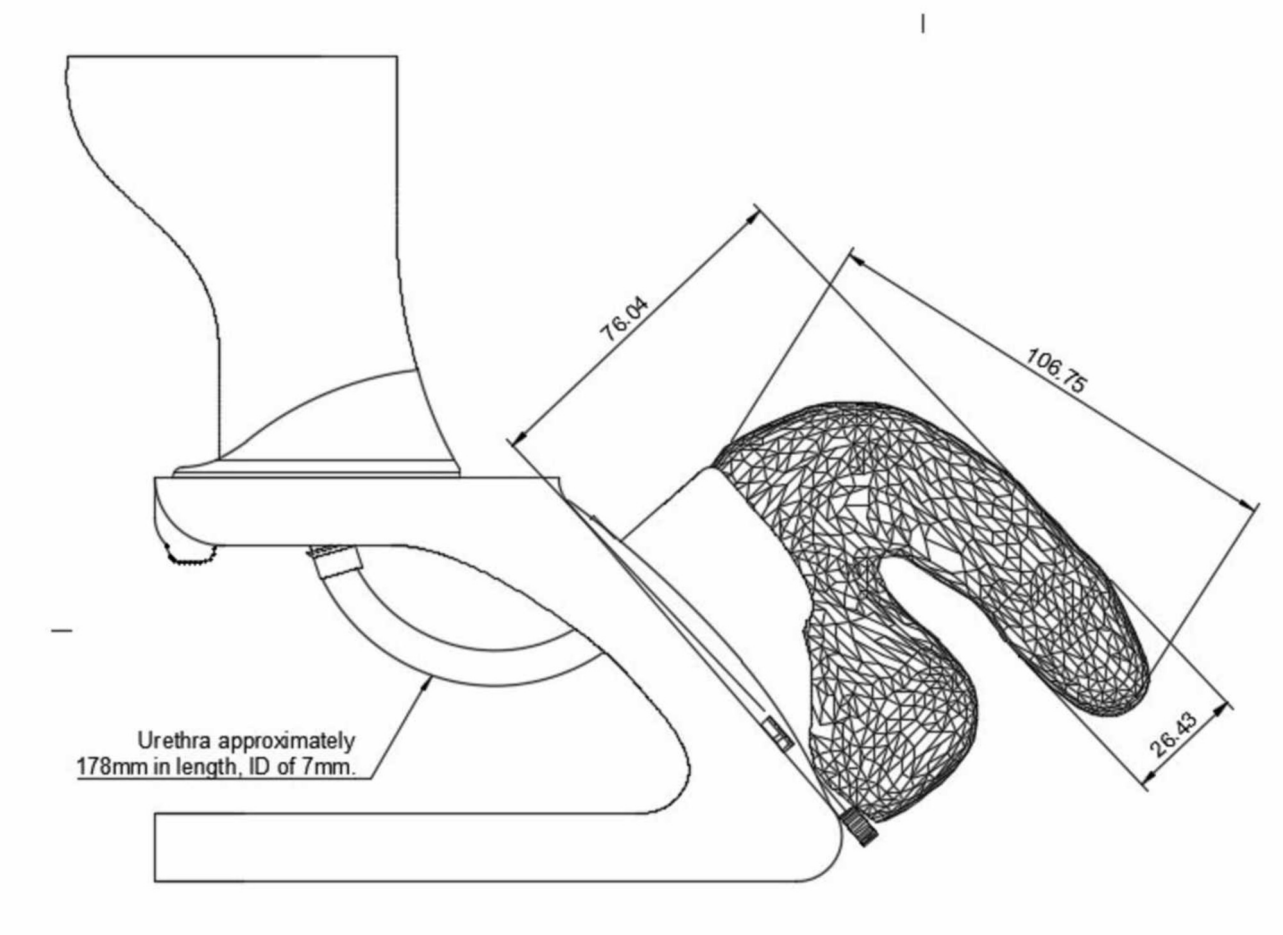
Proposed design for a 3D-printed urinary catheterization model

The model was designed by MunMed3D, a 3D-printing lab based out of the Memorial University School of Medicine, in collaboration with a clinical urologist to ensure anatomical accuracy (Figure 1). This second-generation model was based on an initial design that was previously evaluated by undergraduate medical students who reported increased confidence in the procedure and found the overall simulation beneficial [20]. Feedback from the previous model was incorporated into the design of this iteration.

The model base and bladder were printed with polylactic acid using an Ultimaker 3 (Ultimaker BV, Utrecht, the Netherlands) with a 20% infill and a 0.2 mm layer height. The external genitalia were cast in Smooth-On 00-30 silicone (Smooth-On, Inc., Easton, Pennsylvania). For the urethra and sphincter, a threaded insert and valve body were printed using a Formlabs Form 2 SLA printer (Somerville, Massachusetts) out of Grey Pro resin (Formlabs). The insert was glued into the bottom of the bladder, and the valve body was screwed in to seal against water leakage. A small curved tube with barbs, printed with the fused deposition modeling (FDM) method in polylactic acid, was glued into the bottom of the valve to attach the silicon urethral tube. The front window is made of a 1/8” acrylic sheet and then placed in the frontal aspect of the bladder with silicone caulking. The final cost of the product was estimated to be around $25. 

A total of 64 pre-clerkship undergraduate medical students participated in the study. Students attended a urinary catheter insertion skills station offered at several workshop-style events. The session consisted of a short didactic presentation on catheter insertion as a procedure, including background information on indications, risks, and procedure steps, before the students were given hands-on experience time with the 3D-printed catheter models. The learners were split into small groups of five to six participants and given 10 minutes to practice the steps of catheter insertion, including using the sterile technique, using the catheter models. Following the hands-on session, the students were asked to complete a survey about their experience and comment on some of the anatomical properties of the model, the realism of the procedure, and the overall value of the model as a teaching tool for increasing procedural confidence and competency. Questions were asked on a five-point Likert scale, with one corresponding to “not realistic” or “not recommended to other learners” and a five for “highly realistic” or “highly recommended to other learners.” The questions asked and the results of the survey are outlined in Table 1.

**Table 1:** Participant responses for a 3D-printed urinary catheter simulation model by undergraduate medical students (n = 64)

			n = 64		
First-year students				34 (55.7%)	
Second-year students				26 (42.6%)	
Third-year students				1 (1.6%)	
Unknown year				3 (4.7%)	
Previous training in urinary catheterization, any year				4 (6.3%)	
Physical attributes			Score (1 = “not realistic” to 5 = “highly realistic”)		
	Anatomical structure		4.36 ± 0.68		
	Color		4.36 ± 0.65		
	Shape		4.42 ± 0.64		
	Texture		4.13 ± 0.77		
	Size		4.38 ± 0.60		
	Material		4.25 ± 0.76		
	Overall		4.41 ± 0.58		
Realism of experience			Score (1 = “not realistic” to 5 = “highly realistic”)		
	Stabilizing catheter		4.41 ± 0.59		
	Inserting catheter		4.33 ± 0.70		
	Advancing catheter		4.22 ± 0.73		
	Appearance of urine		4.11 ± 0.96		
	Balloon inflation		4.49 ± 0.59		
			Score (1 = “not at all effective” to 5 = “very effective”)		
Increasing skills-based competency			4.48 ± 0.62		
Increasing confidence			4.40 ± 0.71		
Increasing preparation for real patients			4.15 ± 0.76		
			Percentage of respondents agreeing with the statement		
This model increased my understanding of the procedure					98.4%
This model would be recommended to assist my ongoing education and training					98.4%
This model would be recommended to other trainees					100%
			Score (1 = “not at all relevant” to 5 = “very relevant”)		
Overall value as a training tool			4.62 ± 0.58		

## Results

Following the simulation sessions, surveys were completed by 64 pre-clerkship medical students. Average scores on the 5-point scale were recorded for the assessed domains of physical attributes, realism of experience, and how well the models increased self-assessed competence and confidence in the procedure. The results of the surveys are compiled in Table 1. The percentage of students ranking attributes as a 4 or 5 were summed as an overall measure of student acceptability of the model as being an appropriate simulation, a 4 or 5 corresponding to scores of “realistic” or “highly realistic.” Surveys were collected on three separate simulation events. Of the respondents, 55.7% reported being in their first year of studies, 42.6% were in the second year, and one person was in their third year of training. Only 6.3% of respondents reported previous training in urinary catheter simulation.

## Discussion

**Figure 2:**
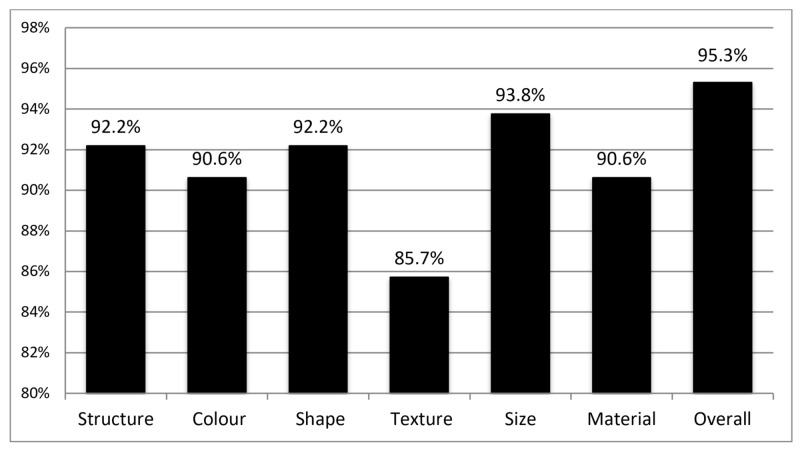
Percentage of respondents who indicated a 4 or 5 (“realistic” or “highly realistic”) for the anatomical domains of the 3D model.

**Figure 3:**
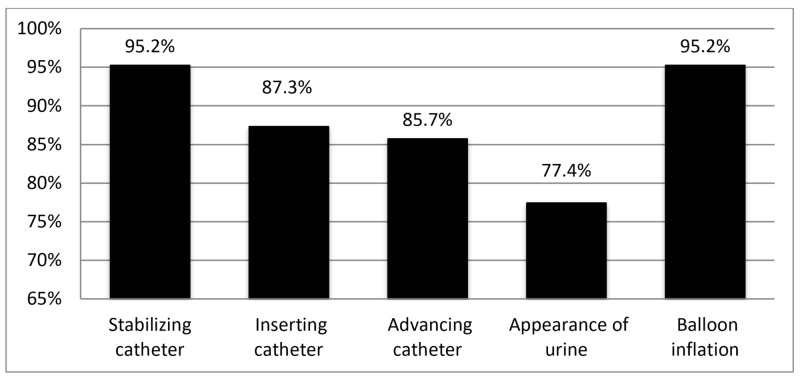
Percentage of respondents who indicated a 4 or 5 (“realistic” or “highly realistic”) for the simulation realism domains of the 3D-model

This study found generally positive feedback surrounding the urinary catheter model, with the majority of participants indicating highly realistic anatomical scores and procedural accuracy, as well as indicating that the model was a useful training tool overall, which would be highly recommended to others. The model was felt to be highly anatomically accurate, with over 90% of respondents indicating realistic (4 or 5 on Likert responses) for six out of seven anatomical domains, as indicated in Figure 2. Similarly, the model was found to be a realistic simulation as outlined in Figure 3.

In comparison to the more traditional pelvis-style models that have been used for catheterization teaching, the 3D-printed models incorporate several unique design features that offer an advantage. They are significantly less expensive, as high fidelity models can reach several hundred dollars. The silicone external genitalia are highly mobile, offering more realistic manipulation than the traditional model. The 3D-printed model allows for the visualization of the urinary tract and bladder, allowing the user to appreciate the function of the catheter in the bladder. Fluid is retained in the bladder using a one-way valve that offers a slight amount of resistance when passing a catheter through the urinary tract, mechanical feedback that is representative of breaching the internal urethral sphincter on a real patient. The bladder fluid then drains through the catheter before the balloon is inflated and held snugly in the bladder neck. The front section of the bladder is transparent, allowing for the visualization of the inflated catheter balloon to appreciate the proper length of insertion and potential for urethral injury. The design of the model features a base design that will allow for the future implementation of a modular design, enabling quick alterations to the model to incorporate pathologic models such as a stricture or enlarged prostate. There are several studies evaluating the effectiveness of urinary catheterization simulation in medical students. Rodriguez-Diez et al. examined the medical students’ confidence levels before and after a structured urinary catheterization simulation module, finding an increase from 15.5% (men) and 10.3% (women) to 87% (men) and 94% (women) [9]. A randomized controlled trial by Zhong et al., featuring a transparent urinary tract simulator demonstrated improved procedural skills in the experimental arm of the study, highlighting the importance of direct visualization in the acquisition of urethral skills [11]. Todsen et al. found that simulation skills were retained at six weeks following simulation training in 64 medical students but did not find that the addition of a training video was helpful in improving performance [23].

This study was designed to be an initial evaluation of a novel catheterization model such that it would show an acceptable level of anatomical accuracy and realism for pre-clerkship training. Using traditional models, it has been shown that learners develop increased confidence with catheterization on a subjective basis. Participants using the 3D-printed urinary catheterization model rated the model as effective in increasing their confidence (4.40±0.71 out of 5). They also felt more competent performing the procedure (4.48±0.62), and that this model helped them prepare for insertion on a real patient (4.15±0.76), findings that have real-world implications of simulation but have not been described using traditional models.

There were several limitations to this study. Due to the subjective nature of the survey assessment, respondents could have felt that any simulation training, regardless of whether it was with this particular model, would be beneficial and scored similarly. However, given the ease and cost of production for the 3D model, increasing the access of simulation training in the situation where there may be large class sizes and reduced exposure to a more traditional model would promote uptake regardless. In certain situations, where students may not have had pre-clerkship exposure to catheterization simulation, the 3D model has the potential to provide an alternative where one may not exist.

Only 6.3% of the learners in this study had previously participated in urinary catheterization, meaning that a subjective assessment of the use of this model by individuals without much exposure to the procedure would carry less weight. As this data was collected at three separate teaching sessions, there may have been variation in the participating students or presentation of the procedure which may have affected the results of the study.

As well, this study was designed as product evaluation for a novel 3D device, and as such makes no attempts to comment on the construct validity of the model in teaching the procedure to an acceptable level as would a validated simulation model. Further studies are required to validate this model and assess whether practicing on such a model allows for fewer complications when catheterization is performed by trainees on patients.

## Conclusions

This 3D-printed urinary catheterization model was designed to enhance learner understanding of the catheter insertion procedure, providing a realistic simulation with anatomical feedback. The model was tested on 64 undergraduate medical learners, who were given a didactic session before using the model to practice catheter insertion. Students felt that the model increased their self-reported confidence and competence in performing urinary catheter insertion, as well as representing a realistic catheter insertion simulation. These models have the potential to be a useful adjunct when teaching urinary catheterization in undergraduate medical learners.
